# Supplementary material for: Forecasting the magnitude of the largest expected earthquake
Source: Nat Commun. 2019 Sep 6;10:4051. doi: 10.1038/s41467-019-11958-4 (PMC6731311; doi:10.1038/s41467-019-11958-4)
Supplement: Supplementary file 1 — Supplementary Inoformation [file 41467_2019_11958_MOESM1_ESM.pdf]

Supplementary Information for:

Forecasting the Magnitude of the Largest Expected Earthquake

Robert Shcherbakov[1,2,*], Jiancang Zhuang[3], Gert Zöller[4], and Yosihiko Ogata[3]

[1]Department of Earth Sciences, University of Western Ontario, London, Ontario, N6A 5B7, Canada
[2]Department of Physics and Astronomy, University of Western Ontario, London, Ontario, N6A 3K7, Canada
[3]Institute of Statistical Mathematics, 10-3 Midori-Cho, Tachikawa-Shi, Tokyo 190-8562, Japan
[4]Institute of Mathematics, University of Potsdam, Potsdam-Golm, 14476, Germany
[*]rshcherb@uwo.ca

Supplementary Figures

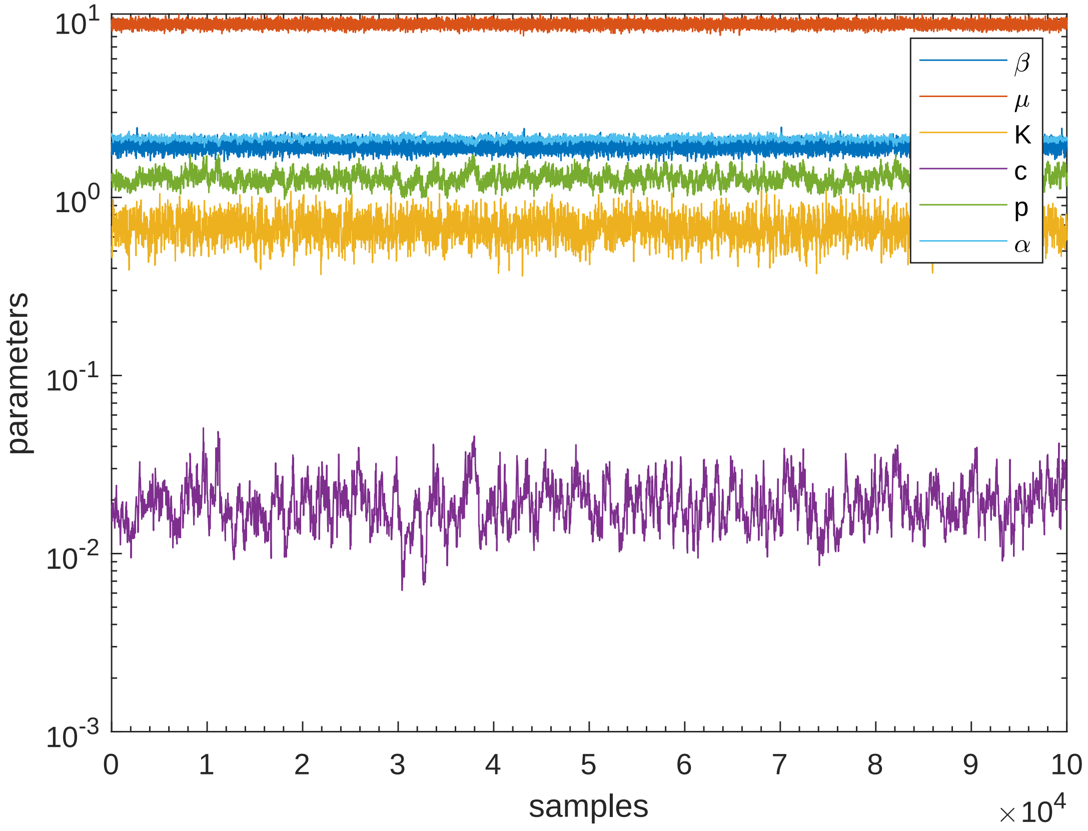

Supplementary Figure 1. The MCMC chains of the ETAS parameters sampled from the posterior distribution, Eq. (3), for the Kumamoto sequence from $T_s = 0.05$ to $T_e = 2.16$ days and for earthquake magnitudes above $m_c = 3.3$. The total number of $200,000$ steps were generated and $100,000$ steps were discarded as burn-in.

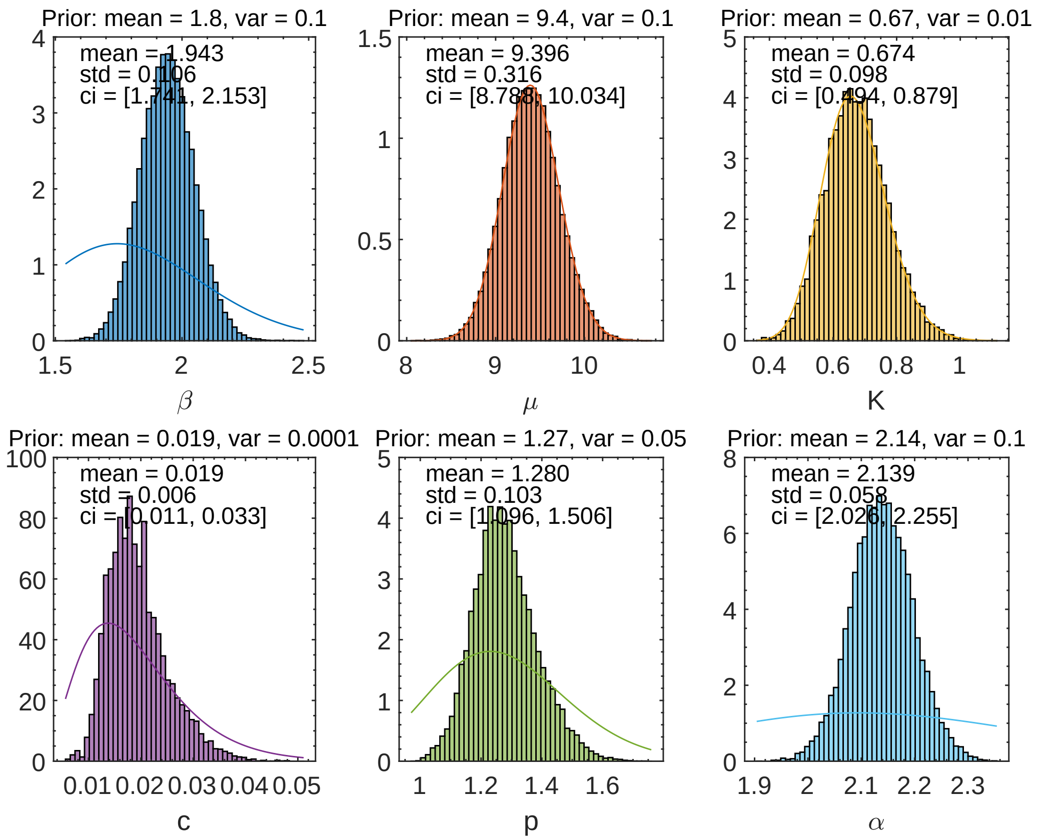

Supplementary Figure 2. The distribution of the ETAS parameters computed from the MCMC chains given in Figure 1. The corresponding mean, standard deviation, and 95% Bayesian confidence bounds for the parameters are provided in the legend. The solid curves represent the prior Gamma distribution for each model parameter.

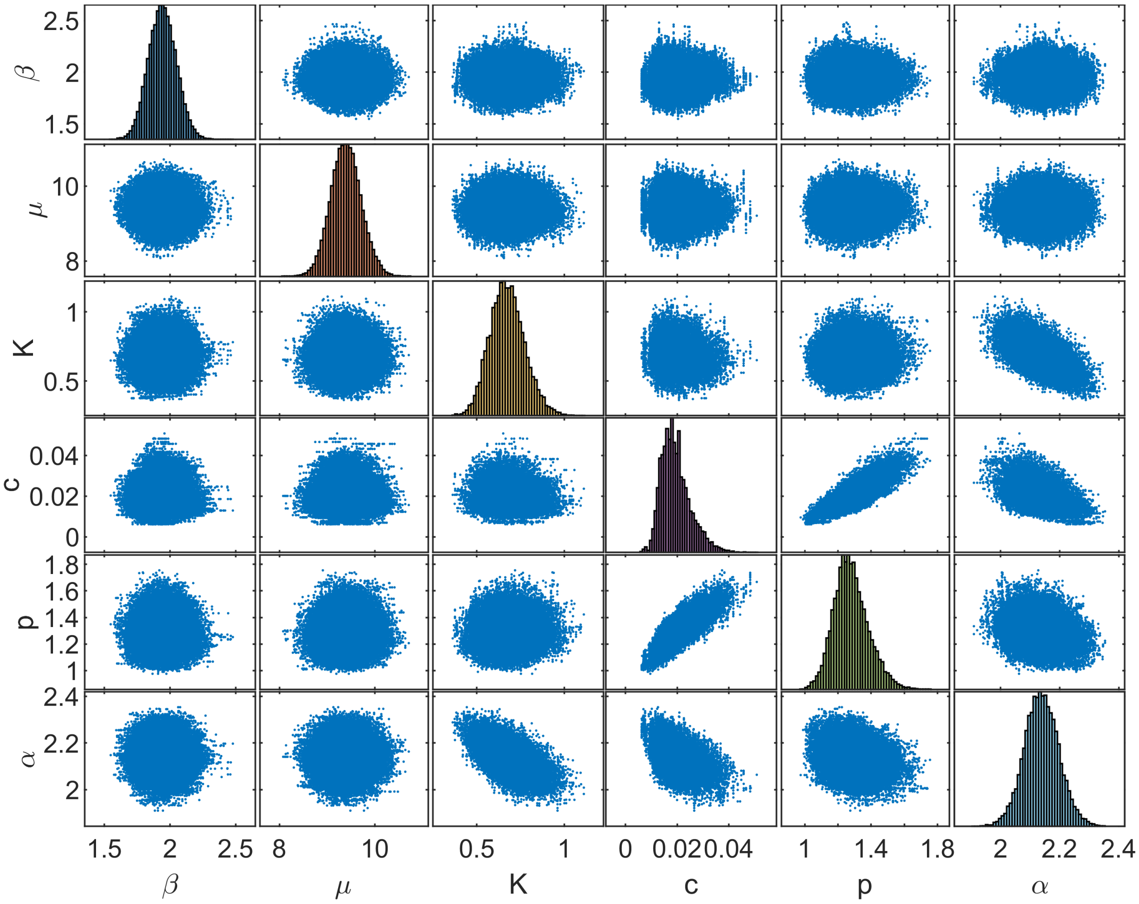

Supplementary Figure 3. The matrix plot of the pairs of the ETAS parameters computed from the MCMC chains given in Figure 1 and showing the correlation structure of the simulated parameters.

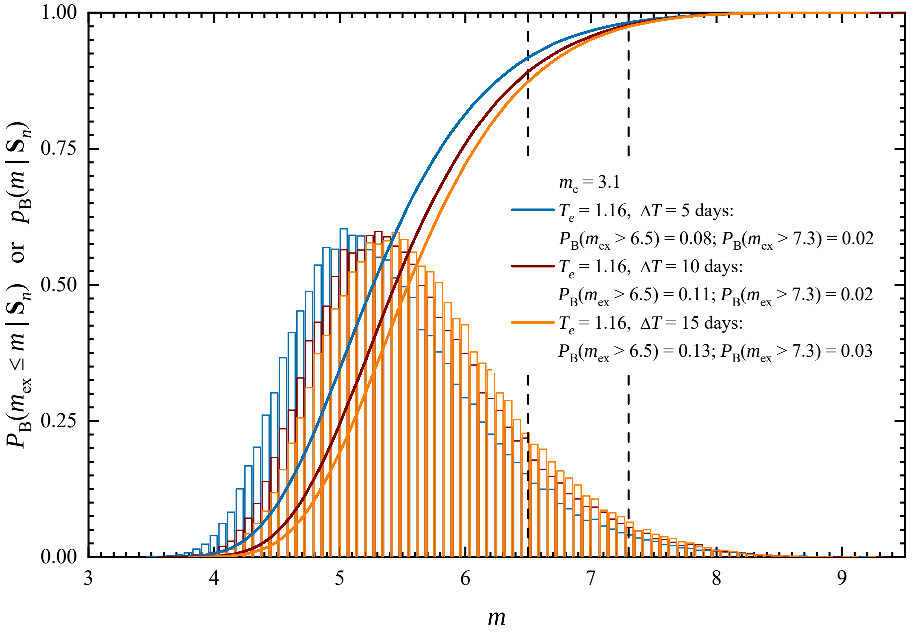

Supplementary Figure 4. Bayesian predictive distribution $P_B(m_{ex} \leq m|\mathbf{S}_n)$ as solid curves and the corresponding probability density function $p_B(m|\mathbf{S}_n)$, as histograms, for the sequence initiated by the April 14, 2016, M6.5 foreshock of the M7.3 Kumamoto earthquake (April 16, 2016). Each curve corresponds to the same early training time interval from $T_s = 0.03$ to $T_e = 1.16$ days with all the events above magnitude $m_c \geq 3.1$ and for the different forecasting time intervals $\Delta T = 5, 10, 15$ days. The probabilities to have large earthquakes above magnitudes $m_{ex} \geq 6.5$ and $m_{ex} \geq 7.3$ are given in the legend.

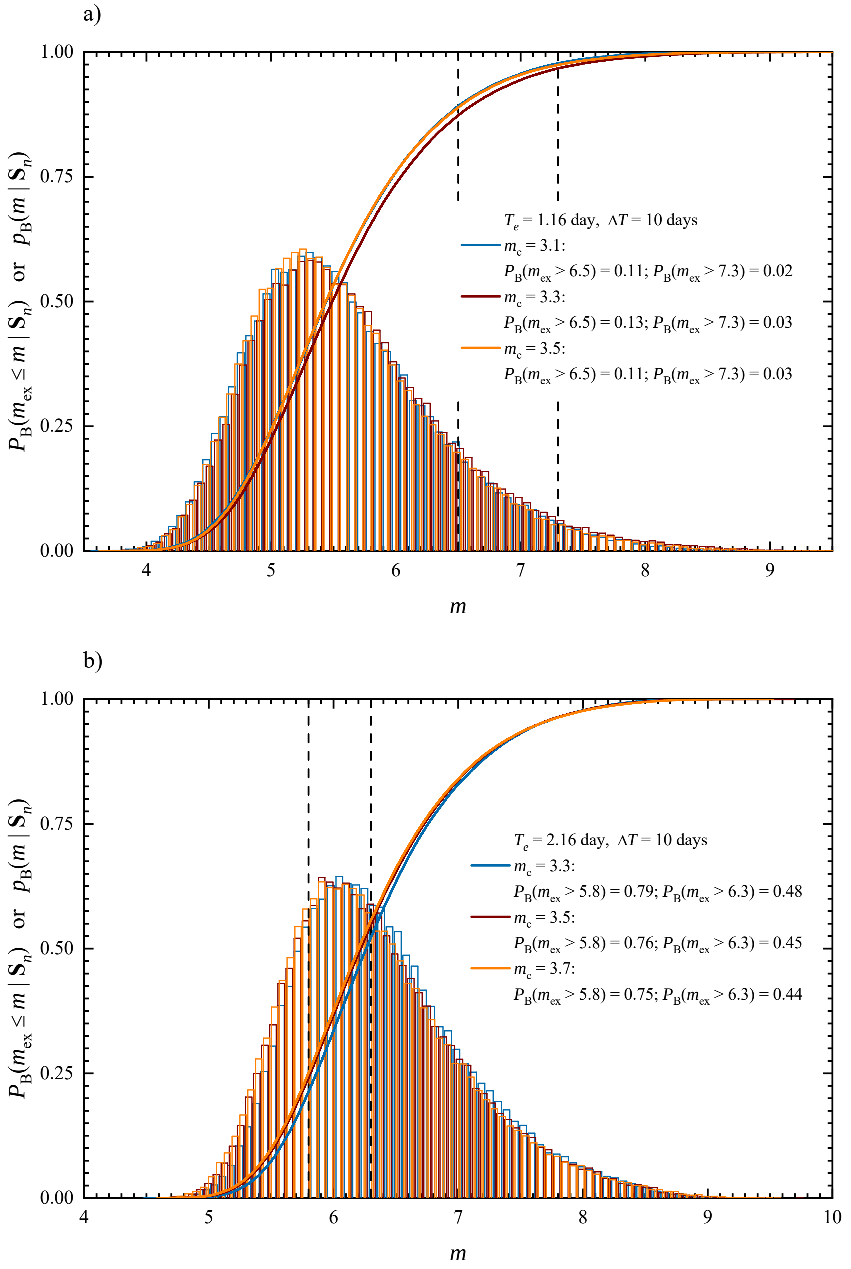

Supplementary Figure 5. Bayesian predictive distribution $P_B(m_{ex} \le m|\mathbf{S}_n)$ as solid curves and the corresponding probability density function $p_B(m|\mathbf{S}_n)$, as histograms, for the sequence initiated by the April 14, 2016, M6.5 foreshock of the M7.3 Kumamoto earthquake (April 16, 2016). Each curve corresponds to the same early training time interval a) from $T_s = 0.03$ to $T_e = 1.16$ days and b) from $T_s = 0.05$ to $T_e = 2.16$ days, a fixed forecasting time interval $\Delta T = 10$ days, and varying lower magnitude cutoffs a) $m_c = 3.1$, 3.3, and 3.5 and b) $m_c = 3.3$, 3.5, and 3.7. The probabilities to have large earthquakes above magnitudes a) $m = 6.5$ and $m = 7.3$ and b) $m = 5.8$ and $m = 6.3$ are given in the legend.

a)

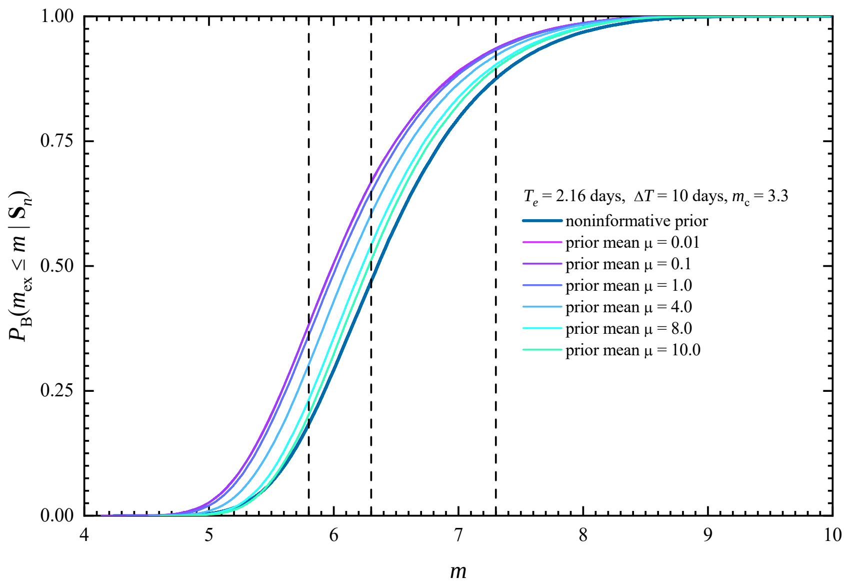

b)

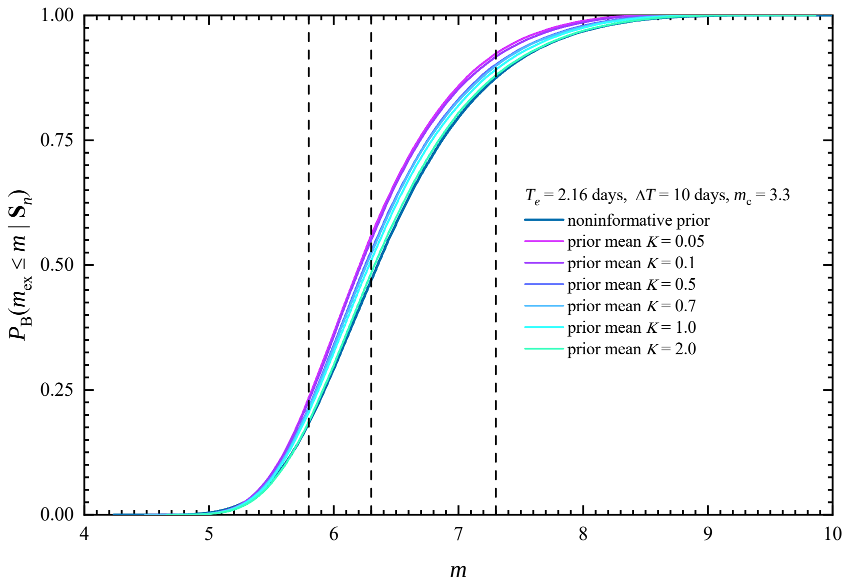

Supplementary Figure 6. Comparison of the Bayesian predictive distributions $P_B(m_{ex} \le m|\mathbf{S}_n)$ for the Kumamoto sequence for varying means of the Gamma prior distribution. Each curve corresponds to the same early training time interval from $T_s = 0.05$ to $T_e = 2.16$ days, a fixed forecasting time interval $\Delta T = 10$ days. a) The mean value of the prior distribution corresponding to the ETAS parameter μ is varied. b) The mean value of the prior distribution corresponding to the ETAS parameter K is varied.

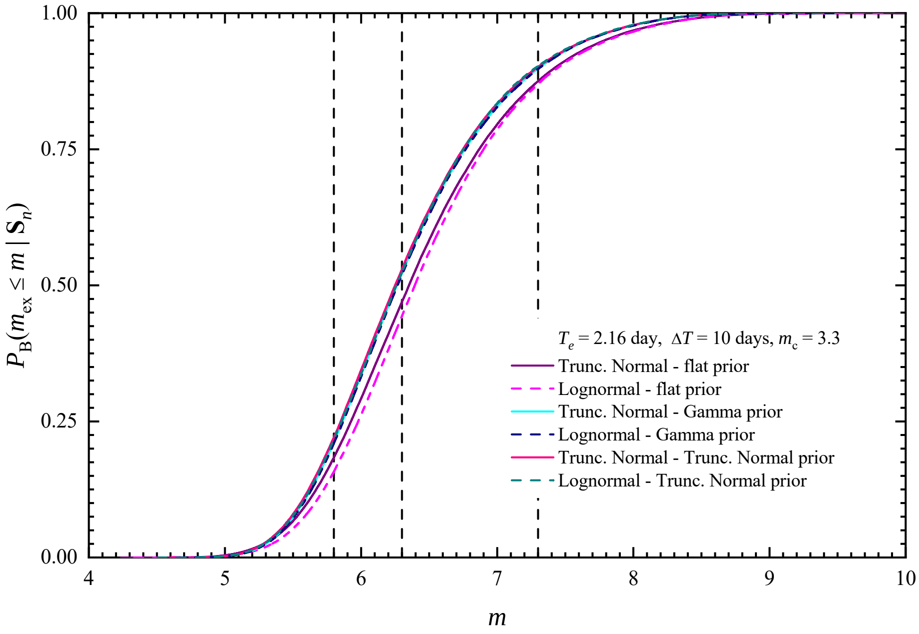

Supplementary Figure 7. Comparison of the Bayesian predictive distributions $P_B(m_{ex} \leq m|\mathbf{S}_n)$ for the largest expected aftershock to be larger than m for several combinations of the proposal and prior distributions. Each curve corresponds to the same training time interval from $T_s = 0.05$ to $T_e = 2.16$ days, a fixed forecasting time interval $\Delta T = 10$ days, and the lower magnitude cutoff $m_c = 3.3$. The distributions were computed using the following pairs for the proposal distribution and the prior distribution: truncated Normal and flat prior (solid blue curve); lognormal and flat prior (dashed magenta curve); truncated Normal and Gamma prior (solid light green curve); lognormal and Gamma prior (dashed blue curve); truncated Normal and truncated Normal prior (solid pink curve); lognormal and truncated Normal prior (dashed violet curve).

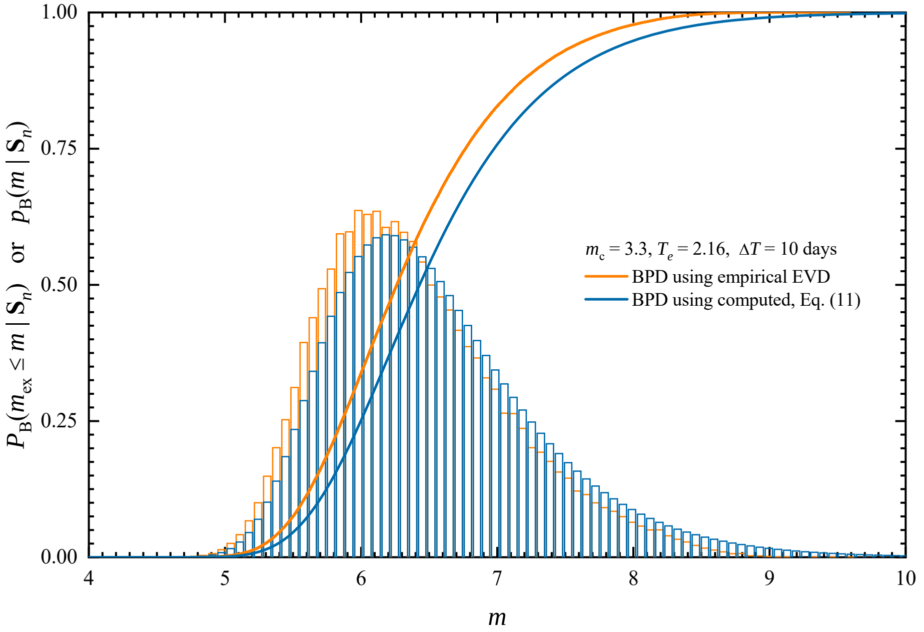

Supplementary Figure 8. Comparison of the extreme value distributions computed using Eq. (11) (solid blue curve) and computed empirically (solid orange curve) by simulating the ETAS model during the forecasting time interval $\Delta T = 10$ days and using the MCMC chain of the model parameters given in Figure S1.

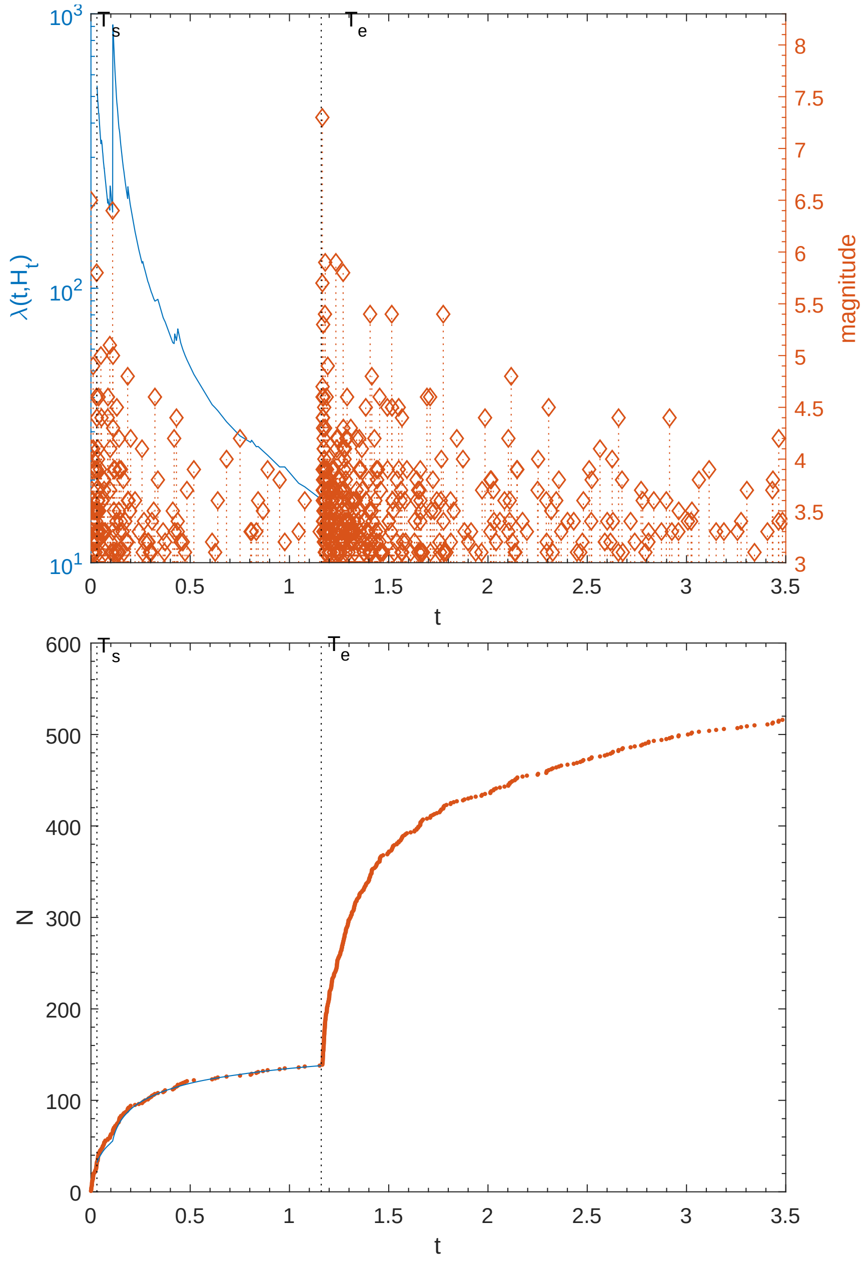

Supplementary Figure 9. The ETAS model fitting to the 2016 Kumamoto, Japan, earthquake sequence for the target time interval $[T_s, T_e] = [0.03, 1.16]$, where $T_0 = 0$ corresponds to the time of the occurrence of M6.5 foreshock. The following ETAS parameters were estimated: $\mu = 1.0$, $K = 0.73$, $c = 0.02$, $p = 1.27$, and $\alpha = 2.1$ for the earthquakes above $m_c = 3.1$. a) The earthquake magnitudes in the sequence and the estimated conditional earthquake rate (solid blue curve) according to the ETAS model, $\lambda_\omega(t|\mathscr{H}_t)$. b) The plot of the cumulative earthquake rate (orange symbols) and the ETAS model fit (solid blue curve). c) The cumulative earthquake rate and the ETAS model fit in transformed time.

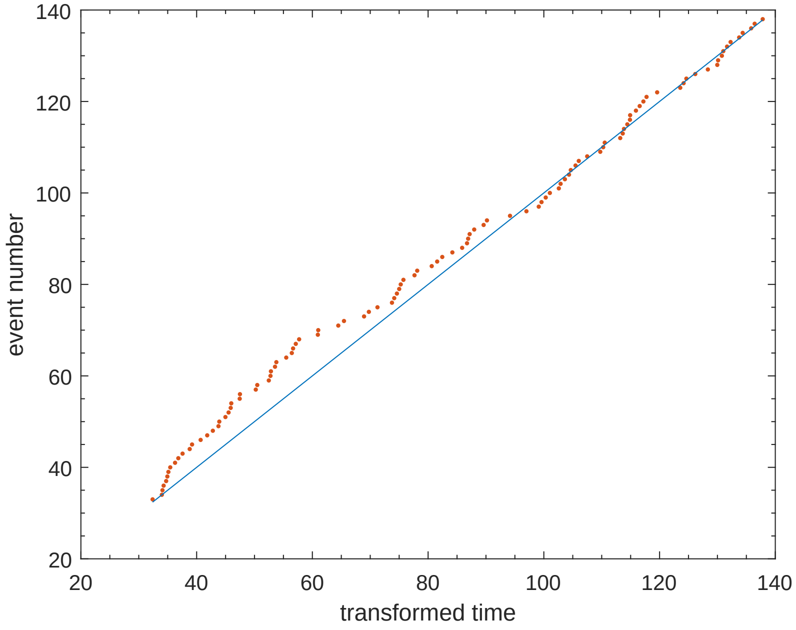

Supplementary Figure 9. (Continued.)

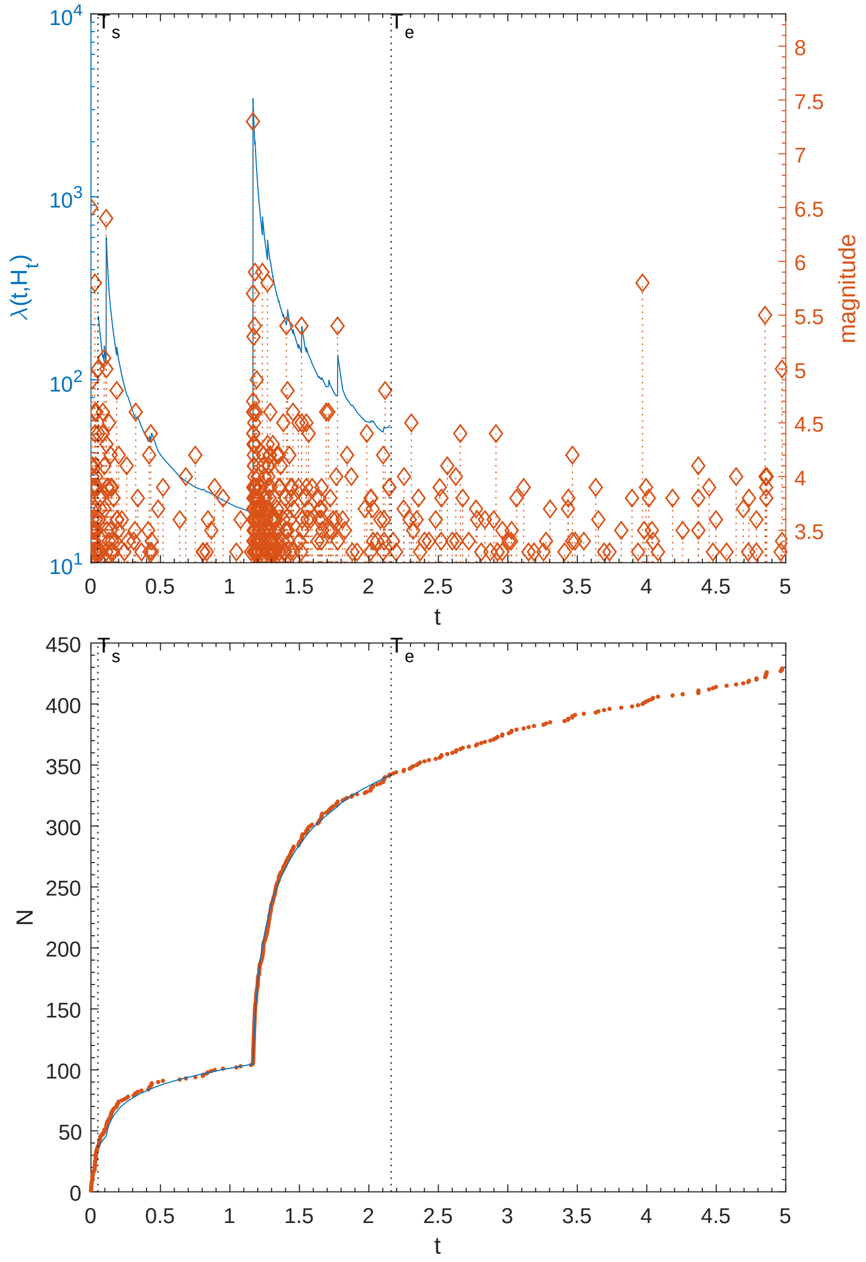

Supplementary Figure 10. The ETAS model fitting to the 2016 Kumamoto, Japan, earthquake sequence for the target time interval $[T_s, T_e] = [0.05, 2.16]$, where $T_0 = 0$ corresponds to the time of the occurrence of M6.5 foreshock. The following ETAS parameters were estimated: $\mu = 9.36$, $K = 0.67$, $c = 0.019$, $p = 1.27$, and $\alpha = 2.14$ for the earthquakes above $m_c = 3.3$. a) The earthquake magnitudes in the sequence and the estimated conditional earthquake rate (solid blue curve) according to the ETAS model, $\lambda_\omega(t|\mathscr{H}_t)$. b) The plot of the cumulative earthquake rate (orange symbols) and the ETAS model fit (solid blue curve). c) The cumulative earthquake rate and the ETAS model fit in transformed time.

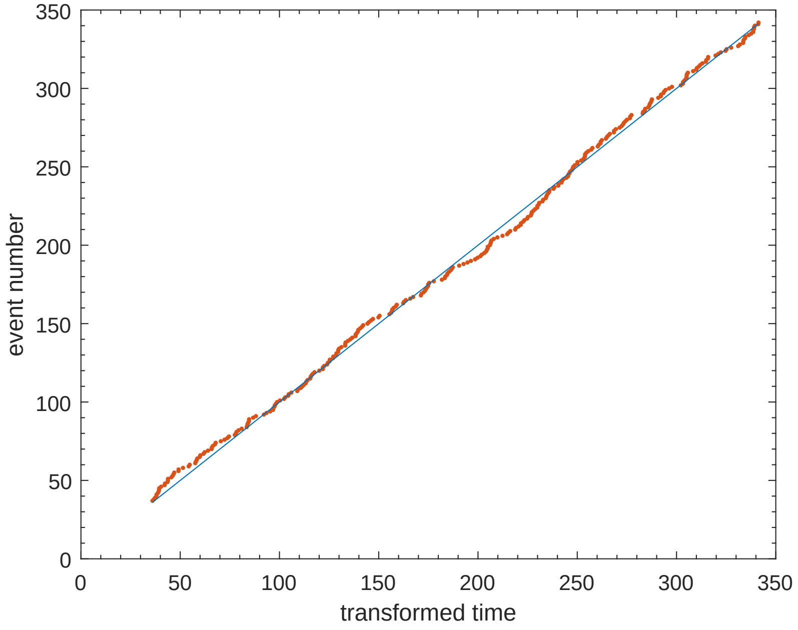

Supplementary Figure 10. (Continued.)

Supplementary Notes

Supplementary Note 1. Analytical derivation of the extreme value distribution for the ETAS model.

Consider the conditional intensity, Eq. (7), of the ETAS model in the following form

$$\lambda(t|\mathscr{H}_t) = \mu + \sum_{i:t_i<t} \kappa(m_i) g(t-t_i)\,, \tag{S1}$$

where

$$\kappa(m) = A\mathrm{e}^{\alpha(m-m_0)} \quad \text{and} \quad g(t) = \frac{p-1}{c}\left(1+\frac{t}{c}\right)^{-p}. \tag{S2}$$

This form of the conditional rate is equivalent to the one given in Eq. (7) with $A = K(p-1)/c$.

Suppose that we are interested in the maximum earthquake magnitude in the forecasting time interval $[T_1,\, T_2]$ under the condition that n earthquakes occurred during the preceding training time interval $[0,\, T_1]$, $\mathbf{S} = \{(t_i, m_i) : i = 1,\, 2,\, ...,\, n\}$. In the following, we derive an expression for this maximum magnitude in a similar fashion as in[1–5].

The probability that the maximum magnitude m_{ex} in $[T_1,\, T_2]$ is

$$\begin{aligned} H(m, T_1, T_2 \mid \mathbf{S}) &= \Pr\left\{ \begin{array}{l} \text{the maximum magnitude } m_{\mathrm{ex}} \\ \text{in } [T_1, T_2] \text{ is less than } m \end{array} \middle| \begin{array}{l} \text{observation history} \\ \mathbf{S} \text{ during } [0,\, T_1] \end{array} \right\} \\ &= P_0 \times \prod_i P_i\,, \end{aligned} \tag{S3}$$

where i runs over all the events in $\mathbf{S}$,

$$P_0 = \Pr\left\{ \begin{array}{l} \text{All background events in } [T_1, T_2] \text{ are less than } m, \\ \text{and any descendant of the above background events} \\ \text{is less than } m \text{ if it occurs in } [T_1, T_2] \end{array} \right\}, \tag{S4}$$

and

$$P_i = \Pr\left\{ \begin{array}{l} \text{Any direct offspring from Event } i \text{ in } \mathbf{S} \text{ is less than } m \text{ if it} \\ \text{occurs in } [T_1, T_2], \text{ and each of the descendants in all} \\ \text{the generations from the direct offspring of Event } i \text{ that are} \\ \text{in } [T_1, T_2] \text{ is less than } m \text{ if it falls in } [T_1, T_2] \end{array} \right\}, \tag{S5}$$

for $i = 1, 2, ..., n$.

Using the total probability theorem, one can obtain,

$$
\begin{aligned}
P_0 &= \sum_{k=0}^{\infty}\left(\Pr\left\{\begin{array}{l}\text{Each background event in } [T_1,T_2] \text{ is less}\\ \text{than } m \text{ and any of its descendants is less}\\ \text{than } m \text{ if it occurs in } [T_1,T_2]\end{array}\,\middle|\,\begin{array}{l}k \text{ background}\\ \text{events occur}\end{array}\right\}\right.\\
&\qquad\left.\times\Pr\left\{\begin{array}{l}k \text{ background}\\ \text{events occur}\end{array}\right\}\right)\\
&= \sum_{k=0}^{\infty}\left[\int_{m_c}^{m}\int_{T_1}^{T_2}\xi(m,T_2-t^*;m^*)\,s(m^*)\,\frac{1}{T_2-T_1}\,\mathrm{d}t^*\,\mathrm{d}m^*\right]^k \frac{\mu^k(T_2-T_1)^k}{k!}\mathrm{e}^{-\mu\,(T_2-T_1)}\\
&= \mathrm{e}^{-\mu\,(T_2-T_1)}\exp\left[1-\frac{1}{T_2-T_1}\int_{m_c}^{m}\int_{T_1}^{T_2}\xi(m,T_2-t^*;m^*)\,s(m^*)\,\mathrm{d}t^*\,\mathrm{d}m^*\right]. \qquad \text{(S6)}
\end{aligned}
$$

In the above equation

$$
\xi(m,\delta t;m^*) = \Pr\left\{\begin{array}{l}\text{For an event occurring at any time, say } t^*,\\ \text{of magnitude } m^*, \text{ each of its descendants in any}\\ \text{generation is less than } m \text{ if it falls in } [t^*,t^*+\delta t]\end{array}\right\}, \qquad \text{(S7)}
$$

which does not depend on t^* because of stationarity of the process. $\frac{1}{T_2-T_1}\int_{m_c}^{m}\int_{T_1}^{T_2}\xi(m,T_2-t^*;m^*)\,s(m^*)\,\mathrm{d}t^*\,\mathrm{d}m^*$ is the probability that any background event in $[T_1,T_2]$ is less than m and has no descendant of magnitude m or higher, and $\frac{1}{T_2-T_1}$ and $s(m)$ are the probability density functions of the occurrence time and magnitude for the background events in $[T_1,T_2]$, respectively. Similarly one can have

$$
\begin{aligned}
P_i &= \sum_{k=0}^{\infty}\left(\Pr\left\{\begin{array}{l}\text{Each direct offspring in } [T_1,T_2]\\ \text{from Event } i \text{ is less than } m \text{ and any}\\ \text{descendant from this direct offspring}\\ \text{is less than } m \text{ if it occurs in } [T_1,\,T_2]\end{array}\,\middle|\,\begin{array}{l}\text{Event } i \text{ produces } k \text{ direct}\\ \text{offsprings in } [T_1,\,T_2]\end{array}\right\}\right.\\
&\qquad\left.\times\Pr\left\{\begin{array}{l}\text{Event } i \text{ produces } k \text{ direct}\\ \text{offsprings in } [T_1,\,T_2]\end{array}\right\}\right)\\
&= \sum_{k=0}^{\infty}\left(\left[\int_{m_c}^{m}\int_{T_1}^{T_2}\xi(m,T_2-t^*;m^*)\,s(m^*)\,g(t^*-t_i)\,\mathrm{d}t^*\,\mathrm{d}m^*\right]^k\right.\\
&\qquad\left.\times\frac{\kappa(m_i)^k[G(T_2-t_i)-G(T_1-t_i)]^k}{k!}\mathrm{e}^{-\kappa(m_i)[G(T_2-t_i)-G(T_1-t_i)]}\right)\\
&= \exp\{-\kappa(m_i)\,[G(T_2-t_i)-G(T_1-t_i)]\}\\
&\qquad\times\exp\left\{\left[1-\int_{m_c}^{m}\int_{T_1}^{T_2}\xi(m,T_2-t^*;m^*)\,s(m^*)\,g(t^*-t_i)\,\mathrm{d}t^*\,\mathrm{d}m^*\right]\right\}, \qquad \text{(S8)}
\end{aligned}
$$

where $G(t)=\int_0^t g(u)\,\mathrm{d}u$. Due to the branching structure of the ETAS model and Eq. (S7), $\xi(m,\delta t;m^*)$

satisfies

$$\xi(m,\delta t;m^*) = \sum_{k=0}^{\infty}\left(\Pr\left\{\begin{array}{l}\text{Each direct offspring in } [t^*,t^*+\delta t]\\ \text{from an event at } (t^*,m^*) \text{ is less}\\ \text{than } m \text{ and any descendant from}\\ \text{this direct offspring is less than}\\ m \text{ if it occurs in } [t^*,t^*+\delta t]\end{array}\middle|\begin{array}{l}\text{An event at } (t^*,m^*)\\ \text{produces } k \text{ direct}\\ \text{offspring in } [t^*,t^*+\delta t]\end{array}\right\}\right.$$

$$\left.\times\Pr\left\{\begin{array}{l}\text{An event at } (t^*,m^*) \text{ produces}\\ k \text{ direct offsprings in } [t^*,t^*+\delta t]\end{array}\right\}\right)$$

$$= \sum_{k=0}^{\infty}\left[\int_{m_c}^{m}\int_0^{\delta t}\xi(m,\delta t-t';m')\,s(m')\,g(t')\,\mathrm{d}t'\,\mathrm{d}m'\right]^k\frac{\kappa(m^*)^k\,G(\delta t)^k}{k!}\mathrm{e}^{-\kappa(m^*)G(\delta t)}$$

$$= \exp\left\{-\kappa(m^*)G(\delta t)\left[1-\int_{m_c}^{m}\int_0^{\delta t}\xi(m,\delta t-t';m')s(m')g(t')\mathrm{d}t'\mathrm{d}m'\right]\right\}. \tag{S9}$$

By inspecting the above equation, Eq. (S9), it is easy to see that

$$\xi(m,\delta t;m^*) = \exp\left[-\kappa(m^*)\,\eta(m,\delta t)\right]. \tag{S10}$$

Substituting Eq. (S10) into Eq. (S9), one obtains

$$\eta(m,\delta t) = G(\delta t)\left[1-\int_{m_c}^{m}\int_0^{\delta t}\exp\left[-\kappa(m')\,\eta(m,\delta t-t')\right]\,s(m')\,g(t')\,\mathrm{d}t'\,\mathrm{d}m'\right], \tag{S11}$$

which is the probability that an arbitrary event has a magnitude less than m and that it produces no descendant equal to m or larger during the future time interval of length δt.

Finally, one obtains

$$P_0 = \exp\left\{-\mu(T_2-T_1)\left[1-\frac{1}{T_2-T_1}\int_{m_c}^{m}\int_{T_1}^{T_2}\mathrm{e}^{-\kappa(m^*)\eta(m,T_2-t^*)}\,s(m^*)\,\mathrm{d}t^*\,\mathrm{d}m^*\right]\right\}, \tag{S12}$$

$$P_i = \mathrm{e}^{-\kappa(m_i)[G(T_2-t_i)-G(T_1-t_i)]}\exp\left[1-\int_{m_c}^{m}\int_{T_1}^{T_2}\mathrm{e}^{-\kappa(m^*)\eta(m,T_2-t^*)}s(m^*)g(t^*-t_i)\mathrm{d}t^*\mathrm{d}m^*\right]. \tag{S13}$$

Therefore, to calculate $H(m,T_1,T_2\mid\mathbf{S})$, one needs to compute the function $\eta(m,\delta t)$ by solving the integral functional equation in Eq. (S11), and then to compute P_0 and P_i in Eqs. (S12) and (S13), respectively. Though such a solution is computationally intensive, it illuminates that the extreme value problems associated with the ETAS model (or a more general Hawkes process) always yield integral functional equations. In practice, it can be replaced by the numerical approximation using the Monte Carlo simulations.

Supplementary Note 2. Fitting the ETAS model to the 2016 Kumamoto, Japan, earthquake sequence

Here we provide the results of the fit of the ETAS model to the 2016 Kumamoto earthquake sequence using the standard maximum likelihood approach. In this analysis the model parameters are estimated by maximizing the log-likelihood function, which can be written as follows:

$$\begin{aligned}\log\left[L\left(\mathbf{S}_{N_e}|\theta,\omega\right)\right] &= -\mu\left(T_e-T_s\right)\\ &-\frac{Kc}{p-1}\sum_{i=1}^{N_s} e^{\alpha(m_i-m_0)}\left[\left(\frac{T_s-t_i}{c}+1\right)^{1-p}-\left(\frac{T_e-t_i}{c}+1\right)^{1-p}\right]\\ &-\frac{Kc}{p-1}\sum_{i=1:T_s\le t_i\le T}^{N_e} e^{\alpha(m_i-m_0)}\left[1-\left(\frac{T-t_i}{c}+1\right)^{1-p}\right]\\ &+\sum_{j=1}^{N_e}\log\left[\mu+K\sum_{i:t_i<t_j}^{N_{t_j}}\frac{e^{\alpha(m_i-m_0)}}{\left(\frac{t_j-t_i}{c}+1\right)^{p}}\right], \end{aligned} \quad \text{(S14)}$$

where N_s is the number of earthquakes in the time interval $[T_0, T_s[$, if any, preceding the target time interval $[T_s, T_e]$. The second sum is over N_e earthquakes that occurred during the target time interval $[T_s, T_e]$. The double sum involves the summation over N_e earthquakes in the interval $[T_s, T_e]$, where the rate in the internal sum is computed for all events starting from T_0 up to a given t_j.

The productivity of the ETAS process during the target time interval $[T_s, T_e]$ is:

$$\begin{aligned}\Lambda_\omega(T_s,T_e) &= \int_{T_s}^{T_e}\lambda_\omega(t|\mathscr{H}_t)\,dt=\int_{T_s}^{T_e}\left[\mu+K\sum_{i:t_i<t}^{N_t}\frac{e^{\alpha(m_i-m_0)}}{\left(\frac{t-t_i}{c}+1\right)^{p}}\right]dt\\ &= \mu\left(T_e-T_s\right)+\frac{Kc}{p-1}\sum_{i=1}^{N_s} e^{\alpha(m_i-m_0)}\left[\left(\frac{T_s-t_i}{c}+1\right)^{1-p}-\left(\frac{T_e-t_i}{c}+1\right)^{1-p}\right]\\ &+\frac{Kc}{p-1}\sum_{i=1:T_s\le t_i\le T_e}^{N_e} e^{\alpha(m_i-m_0)}\left[1-\left(\frac{T_e-t_i}{c}+1\right)^{1-p}\right], \end{aligned} \quad \text{(S15)}$$

where N_s is the number of earthquakes in the time interval $[T_0, T_s[$, if any, preceding the target interval $[T_s, T_e]$. The second sum is over N_e earthquakes that occurred during the target time interval $[T_s, T_e]$. The productivity of the ETAS process $\Lambda_\omega(\Delta T)$ during the forecasting time interval $[T_e, T_e+\Delta T]$ cannot be directly computed and needs to be estimated from the stochastic simulation of the ETAS model due to its intrinsic randomness.

The sequence starting from the M6.5 foreshock that occurred on 14 April 2016 (12:26 UTC) was considered. The starting time $T_0 = 0$ was set to the time of the occurrence of this foreshock. Earthquakes during two target time intervals $[T_s, T_e] = [0.03, 1.16]$ days and $[T_s, T_e] = [0.05, 2.16]$ days were considered to be fitted by the ETAS model. The estimated conditional rate, $\lambda_\omega(t|\mathscr{H}_t)$, and the earthquake magnitudes as marks above the lower cutoff $m_c = 3.1$ are plotted in Supplementary Figure 9a for the first target interval $[T_s, T_e] = [0.03, 1.16]$. When estimating the parameters the constraint $\mu = 0$ was used. The cumulative number of earthquakes above $m_c = 3.1$ is plotted in Supplementary Figure 9b and the earthquake number in the sequence (cumulative number) versus the transformed time is plotted in Supplementary Figure 9c. The transformed time is defined as follows $\tilde{t} \equiv \Lambda_\omega(T_s, t) = \int_{T_s}^{t} \lambda_\omega(t'|\mathscr{H}_{t'})\, dt'$. It is computed by using the estimated ETAS parameters. The plot in transformed time provides visual check on the goodness of fit of the ETAS model, i.e. closer the cumulative earthquake number to the straight line better the ETAS fit is.

Similarly, we provide the estimated ETAS parameters for the second sequence during the target time interval $[T_s, T_e] = [0.05, 2.16]$. The conditional earthquake rate, the earthquake magnitudes, the cumulative rate and the rate in transformed time for this sequence with the lower magnitude cutoff $m_c = 3.3$ are plotted in Supplementary Figure 10.

Supplementary References

1. Saichev, A. & Sornette, D. Distribution of the largest aftershocks in branching models of triggered seismicity: Theory of the universal Båth law. *Phys. Rev. E* **71**, 056127 (2005).

2. Zhuang, J. & Ogata, Y. Properties of the probability distribution associated with the largest event in an earthquake cluster and their implications to foreshocks. *Phys. Rev. E* **73**, 046134 (2006).

3. Vere-Jones, D. A limit theorem with application to Båth's law in seismology. *Adv. Appl. Prob.* **40**, 882–896 (2008).

4. Vere-Jones, D. & Zhuang, J. Distribution of the largest event in the critical epidemic-type aftershock-sequence model. *Phys. Rev. E* **78**, 047102 (2008).

5. Luo, J. W. & Zhuang, J. Three regimes of the distribution of the largest event in the critical ETAS model. *Bull. Seismol. Soc. Am.* **106**, 1364–1369 (2016).
